# Translative lens-based full-field coherent X-ray imaging

**DOI:** 10.1107/S1600577519013742

**Published:** 2020-01-01

**Authors:** Carsten Detlefs, Mario Alejandro Beltran, Jean-Pierre Guigay, Hugh Simons

**Affiliations:** a European Synchrotron Radiation Facility, BP 220, F-38043 Grenoble Cedex, France; b Department of Physics, Technical University of Denmark, 2800 Kgs Lyngby, Denmark

**Keywords:** coherence, imaging, microscopy

## Abstract

A full-field coherent imaging approach suitable for hard X-rays based on a classical (*i.e.* Galilean) X-ray microscope is described. The method combines a series of low-resolution images acquired at different transverse lens positions into a single high-resolution image, overcoming the spatial resolution limit set by the numerical aperture of the objective lens. The optical principles of the approach are described, the successful reconstruction of simulated phantom data is demonstrated, and aspects of the reconstruction are discussed. The authors believe that this approach offers some potential benefits over conventional scanning X-ray ptychography in terms of spatial bandwidth and radiation dose rate.

## Abstract 2

A description and simulation of a full-field coherent imaging approach suitable for hard X-rays based on a classical (*i.e.* Galilean) X-ray microscope.

## 1. Introduction   

Lens-based full-field X-ray microscopy, in which an objective lens between the object and detector creates a magnified image of the object, offers the possibility to image extended objects in a single acquisition. As such, it is well suited for investigating dynamic processes, such as in materials (Snigireva *et al.*, 2018 ▸), chemical reactions (Meirer *et al.*, 2011 ▸) and biological systems (Meyer-Ilse *et al.*, 2001 ▸). However, the spatial resolution of a lens-based full-field microscope is physically limited by the finite numerical aperture (NA) of its objective lens, which tends to be small (0.01 or less) at hard X-ray energies (*E* > 15 keV). Recent developments in X-ray optics have yielded substantial improvements in NA (Schroer & Lengeler, 2005 ▸; Morgan *et al.*, 2015 ▸; Mohacsi *et al.*, 2017 ▸; Matsuyama *et al.*, 2019 ▸), but often at the cost of reducing the working distance to an impractical degree.

Synthetic aperture microscopy offers an alternative route to increasing the NA. One such approach is Fourier ptychographic microscopy (FPM) (Zheng *et al.*, 2013 ▸), which involves combining a series of low-resolution intensity images in Fourier space and subsequently back-propagating to the object plane to recover the exit surface complex wavefield. Varying the angle of the incident full-field illumination samples a wider range of scattering directions, thus improving the space-bandwidth product without the need to move the sample, objective lens or detector (Lohmann *et al.*, 1996 ▸). FPM’s image recovery procedure therefore differs from that of conventional X-ray ptychography (for example, see Rodenburg & Bates, 1992 ▸; Faulkner & Rodenburg, 2004 ▸; Rodenburg & Faulkner, 2004 ▸; Rodenburg *et al.*, 2007 ▸; Thibault *et al.*, 2008 ▸; Maiden & Rodenburg, 2009 ▸; Dierolf *et al.*, 2010 ▸; Maiden *et al.*, 2010 ▸; Humphry *et al.*, 2012 ▸) in that the object support constraints are imposed in Fourier space rather than real space. The original implementation of FPM used a conventional optical microscope (*i.e.* visible light) with a small magnification (2× objective) and NA (0.08) to achieve a synthetic NA of 0.5, resulting in a spatial resolution comparable with a 20× objective while maintaining the much larger field of view and depth of field of the original low-magnification configuration.

Adapting FPM to the X-ray regime could potentially address two key shortcomings of lens-based full-field X-ray microscopy: the compound image corresponds to a larger, synthetic NA, while digital wavefront correction may be used during the image recovery procedure to compensate for lens aberrations (which may be appreciable) (Koch *et al.*, 2016 ▸). Furthermore, as this recovery procedure yields a complex image, one could exploit the phase contrast to dramatically increase sensitivity to weakly interacting objects (Förster *et al.*, 1980 ▸; Snigirev *et al.*, 1995 ▸; Cloetens *et al.*, 1997 ▸). A practical X-ray implementation of FPM requires subtle differences to the original approach, however, since at large-scale facilities (*e.g.* synchrotrons) one cannot directly rotate the incident beam angle in the manner originally proposed by Zheng *et al.* (2013 ▸). Very recently, however, Wakonig *et al.* (2019 ▸) experimentally demonstrated FPM in the X-ray regime by moving a pinhole positioned at the aperture of a condensing lens (Wakonig *et al.*, 2019 ▸), thus steering the incident beam angle at the sample position. Here we describe an alternative approach, where we instead move the lens, collecting images at various overlapping regions. Crucially, the lens and detector are moved transversely to the optical axis in order to avoid the mechanical complexity and imprecision associated with coupled translations/rotations. This approach is conceptually similar to downstream pinhole-scanning methods (*e.g.* Tsai *et al.*, 2016 ▸; Faulkner & Rodenburg, 2004 ▸; Guizar-Sicairos & Fienup, 2008 ▸), albeit using a focusing lens instead of a pinhole.

**Figure 1:**
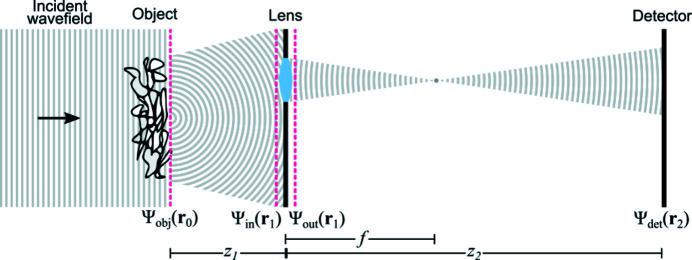
Schematic of the lens translation imaging (LTI) setup.

In this paper, the theory and methodology outlining the idea of lens translation imaging (LTI) is structured as follows. Section 2 describes the image formation problem via mathematical formalisms pertinent to scalar coherent wavefield propagation. The LTI image acquisition method is depicted in Section 3 and accompanied with numerical simulation examples. In Section 4 we detail the iterative phase-retrieval process that reconstructs the wavefield of the exit surface of the imaged object (see Fig. 1 ▸). Results from numerical simulations are also shown.

## 2. Theory of image formation (forward problem)   

Fig. 1 ▸ illustrates the LTI imaging configuration. Monochromatic coherent X-ray plane-wave electromagnetic illumination with wavelength λ propagates rightwards along the optical axis *z*. The incident radiation traverses an object or specimen where the intensity and phase changes incurred are imprinted on the complex wavefield 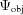 exiting the object. This exit field then propagates downstream a distance 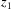 reaching the entry plane 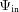 of an optically thin converging lens with finite aperture size and focal length *f*. The field transmitted through the lens 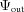 propagates a further distance 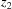 to give 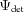, where a spatially sensitive detector is placed that measures the square modulus (intensity) of the wavefield 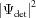, thus excluding all phase information.

We derive an analytical expression for the wavefield 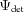 utilizing the linear operator theory of imaging (Nazarathy & Shamir, 1980 ▸). The formalism treats the propagation and passage of optical wavefields though a system as a linear operator acting on some input to yield an associated output. This enables the problem in Fig. 1 ▸ to be undertaken in a ‘cascading’ approach, resulting in the following expression for the wavefield at the detector plane,
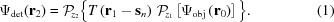
Here, 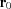 = 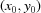, 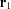 = 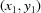 and 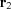 = 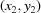 are the Cartesian coordinates normal to *z* corresponding to the object (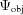), lens (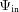 and 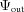) and detector plane (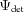), respectively. Note that all *x* and *y* axes at each plane are assumed to be parallel to each other. 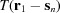 is the transmission function of the lens, where the vector 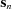 = 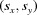 with magnitude 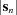 represents the translation position of the center of the lens in relation to the 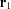 plane. The index *n* is an integer used as an indicator of the lens position.

The operator 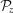, which forward propagates a complex wavefield by a distance *z*, is the operator form of the Fresnel diffraction integral (Goodman, 2005 ▸; Paganin, 2006 ▸; Born & Wolf, 1999 ▸),
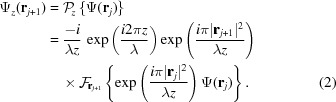
This operator maps a field in a plane defined by the coordinates 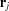 = 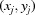 onto a propagated field defined by the coordinates 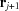 = 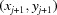, where *j* takes on non-negative integer values (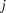 = 0, 1). For example, 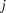 = 0 would correspond to the field mapping of 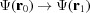. The operation acts from right to left as follows: (i) multiply the input wavefield by a quadratic phase factor in 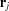; (ii) take the ‘scaled’ Fourier transform which projects a complex function from 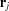 to 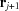; (iii) multiply the result by the quadratic phase factor in 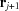 and the constant complex phase shift set by *z*. The Fourier transform convention used here is
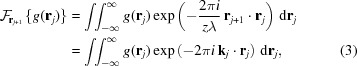
where
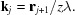
We note the use of the term ‘scaled’, as the Fourier transform used here differs slightly from a conventional Fourier transform in that it maps a complex function from real space onto another complex function also in real space that is re-sampled by a scale factor 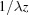 (Goodman, 2005 ▸; Paganin, 2006 ▸).

The complex transmission function of the lens denoted by 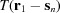 can be decomposed into four separate functions corresponding to the phase shift 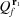, absorption 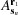, lens aberration 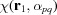 and masking 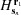 (due to the finite aperture size) of the transmitted wavefield. That is,
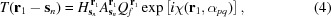
where
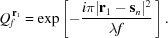
The complex function 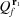 quantifies the phase shifts imparted by the, assumed to be thin, lens on the entering wavefield 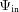 (Paganin, 2006 ▸). These phase shifts are consistent with the condition needed to create focus fields, where the phase exiting the surface of the lens must be such that a spherical wave is collapsed towards a point (Paganin, 2006 ▸). The amplitude attenuation suffered by 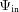 is determined by the function 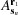. It is important to note that in this section the amplitude function is kept arbitrary in order to accommodate for the various types of X-ray focusing elements that exist. However, for the simulation shown in Section 3 this function takes on the form of a Gaussian distribution corresponding to refractive optics such as compound refractive lenses (CRLs) (Simons *et al.*, 2017 ▸). 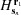 represents the finite aperture size of the lens and serves to transmit only the spatial frequencies of the wavefield 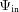, within the radius of the physical aperture (Simons *et al.*, 2017 ▸). 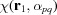 is the aberration function of the lens, which is characterized by the *aberration coefficients* 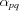, where *p* and *q* are non-negative integers representing the aberration order (Born & Wolf, 1999 ▸). In the case of an ideal thin lens, as is considered in this study, we assume zero aberrations are present [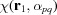 = 0]. Note, however, that in practical settings these lens aberrations need to be either corrected using aberration balancing techniques or iteratively refined in the phase retrieval process – similar to the determination of the illumination function in classical ptychography. This, however, is beyond the scope of this work.

Given that all terms and symbols have been defined in operator form, equation (1) can now be expressed as
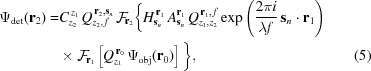
where
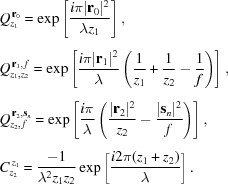
Equation (5) is generally applicable to all systems in which a lens is placed between the object and detector. This includes two special cases:

*The Fourier transforming condition*, where the object is placed very close to the lens plane (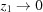) such that 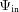 = 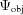, the detector is placed in the back focal plane (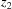 = 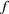) and the lens is completely transparent (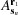 = 1). In this configuration, the measured intensity of the ‘focused field’ becomes the squared modulus of the Fourier transform of object field (*i.e.* 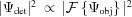). This results in a variation of coherent diffraction imaging in which the lens can be used to reduce the large propagation distances necessary to achieve far-field diffraction patterns in the short-wavelength regime (Quiney *et al.*, 2006 ▸).

*The imaging condition* – considered in this study – corresponds to where the object, lens and detector are placed according to the famous thin lens formula,
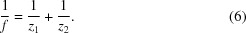
In this condition the term 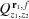 becomes unity, substantially simplifying equation (5) in the context of the forward problem. More importantly, the detected image will resemble an inverted version of the object’s exit surface (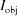) – a valuable asset that will be exploited in the inverse problem described in Section 4.

## 3. Methodology of LTI   

This section describes the image acquisition method for LTI. Returning our attention to the lens plane 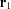 in Fig. 1 ▸, one sees that the finite size of the lens aperture means that a single 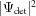 measurement will only register information corresponding to a limited region of 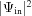. Therefore, acquiring multiple 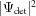 measurements at different lens translation positions becomes paramount if one wishes to record a higher portion of spatial frequency data (real and complex) and subsequently improve the spatial resolution of the compound image.

**Figure 2:**
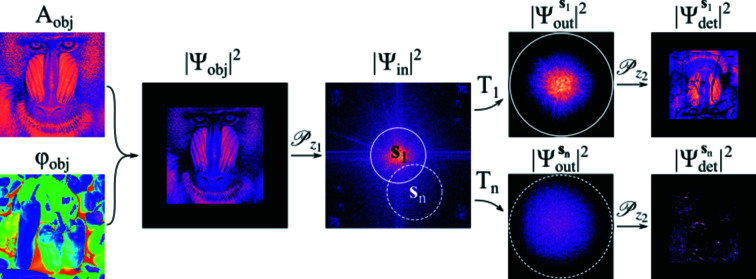
Forward simulations and an illustration of the LTI data acquisition process, from the input amplitude and phase (left), to the intensity at the lens plane (center), to the resulting intensity on the detector (far right). Amplitude and intensity images are scaled from 0 to 1, while phase images are scaled from 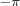 to 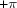. Note that the images include a padding area of zeros around their perimeter.

Fig. 2 ▸ depicts a flow-chart for the LTI methodology, in which a complex test image (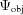) is successively forward propagated to the lens plane (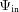 and 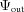) and to the detector plane (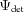). Importantly, the schematic embodies the key idea behind LTI where the lens is translated to different position 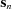 in a way that several overlapping areas of 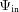 are imaged at the detector.

The forward simulations shown in Fig. 2 ▸ were chosen to be representative of a typical full-field transmission X-ray microscope operating at hard X-ray energies (Snigireva *et al.*, 2018 ▸) and with an X-ray magnification of approximately 18×. The complex object wavefield [*i.e.* 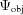 = 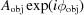, see far left of Fig. 2 ▸] consisted of standard test images of a mandrill and peppers for the amplitude and phase, respectively. The physical size of this wavefield was 25.6 µm (W) × 25.6 µm (H), and the wavelength was chosen to be 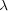 = 0.75 Å, corresponding to a photon energy of 16.5 keV. This field is propagated by a distance 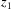 = 0.264 m where the output field 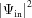 according to the Fresnel number 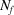 = 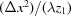 ≃ 10^−4^ lies in the far-field regime for the given object pixel size of 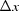 = 50 nm (not to be confused with the detector pixel size of ∼0.9 µm). For a focus distance 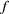 = 0.25 m the detector distance will be 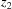 = 4.736 m to satisfy the condition in equation (6) yielding a geometrical magnification of *M* = 17.9. To approximate experimental conditions, the detected intensity images incorporated Poisson noise, which varied from approximately 1.5 to 20% from the central to the outermost lens position. We note that the signal-to-noise is expected to decrease as the corresponding image intensity decreases towards the most distant lens positions from the central axis 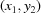 = (0, 0). The attenuation properties of the lens were approximated by an apodized Gaussian distribution,
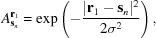
with a variance of 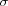 = 25 µm and a physical aperture of radius 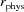 = 75 µm, which is typical for commercially produced two-dimensional Be-based CRLs with this focal length and energy (Simons *et al.*, 2017 ▸).

The far right of Fig. 2 ▸ shows two examples of simulated detected intensity images, labeled as corresponding to lens positions 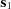 and 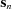. As equation (5) predicts, the intensity corresponding to the central axis position, 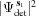, is approximately an inversion of 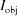. The resolution, however, is considerably poorer due to the masking of high spatial frequencies outside the aperture of 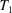. Furthermore, residual features from sharp gradients in the phase map are visible as mild intensity variations in this centered image (Zernike, 1942 ▸). The off-centered image 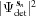 corresponds to a region of 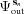 with mostly higher spatial frequency data. The intensity variations in this image thus reveal a higher fraction of morphological detail associated with the phase map, with visible features similar to those seen in differential inference contrast images (Kaulich *et al.*, 2002 ▸; Ou *et al.*, 2013 ▸). This type of contrast is typical in images attained using X-ray imaging techniques such as diffraction-enhanced imaging (Förster *et al.*, 1980 ▸), grating-based interferometry (Pfeiffer *et al.*, 2006 ▸) and speckle-based phase contrast (Morgan *et al.*, 2012 ▸; Bérujon *et al.*, 2012 ▸).

## 4. Iterative phase-retrieval (inverse problem)   

The iterative phase-retrieval aims to recover the object wavefield [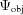 = 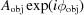] from a series of spatially overlapped translated images, each of which has only amplitude information.

**Figure 3:**
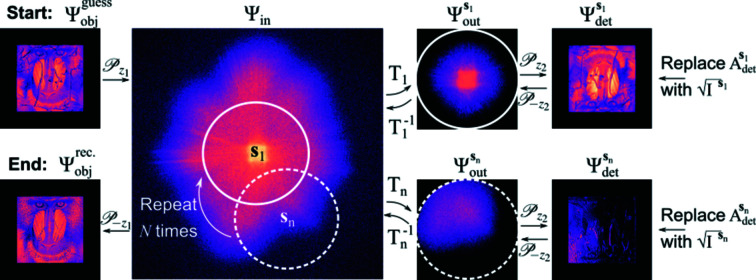
Flow-chart illustrating the iterative phase-retrieval procedure used in LTI.

We explain the phase-retrieval procedure with the aid of Fig. 3 ▸. While only two 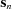 positions are used for explanatory reasons, it can be trivially generalized to arbitrarily many positions (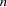 > 2). The procedure (described here in the case of 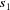) is as follows. (i) Make an initial guess of the object wavefield 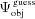. (ii) Forward propagate 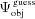 by a distance 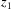 using equation (2) to obtain 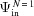. (iii) Multiply 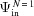 by the lens transmission function for the position 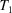 to give 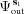. (iv) Forward propagate 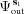 by a distance 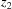 to determine 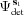. (v) Replace the ampitude with the square root of the measured intensity at that position. (vi) Back propagate by a distance 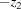 to give an updated 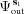. (vii) Update 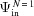 in the area corresponding to position 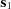 using the extended ptychographic iterative engine (E-PIE) approach of Maiden & Rodenburg (2009 ▸),
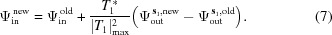
(viii) Move to the neighboring position 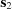 and repeat steps (iii)–(vii). This process [steps (i)–(viii)] is then carried out up to 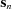 and repeated for *N* iterations or until the error metric 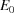 has reached a minimum value. The error metric used here is defined as (Maiden & Rodenburg, 2009 ▸)
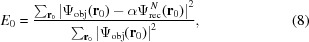
where
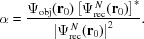
Here, 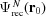 is the reconstructed object wavefield after a particular iteration *N*. The final step (ix) involves back propagating the *N*th iteration of 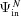 by a distance 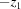, therefore fully recovering 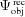.

**Figure 4:**
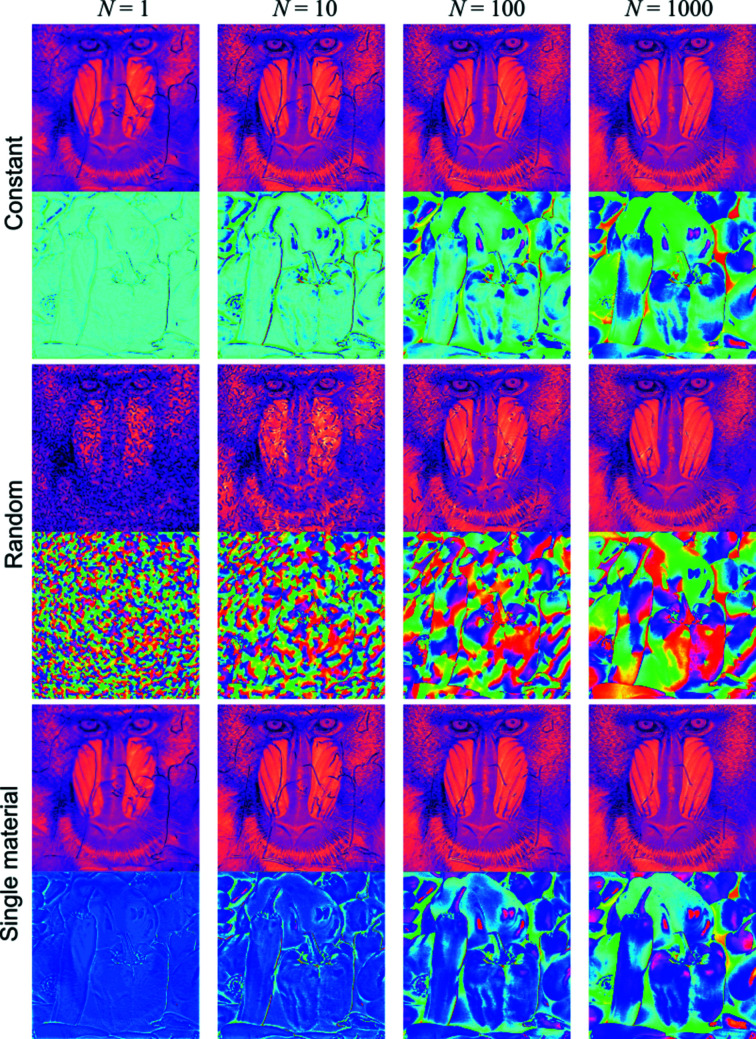
Reconstruction of the object’s wavefield performed with 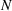 = 1, 10, 100 and 1000 iterations.

The reconstruction procedure was applied to a series of 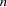 = 25 intensity images calculated for 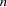 = 25 different lens positions corresponding to a 2.38× increase in NA with an average overlap of 80% of their physical aperture (radius 150 µm). The initial guess utilized the centered intensity measurement as the initial guess of the object’s amplitude 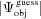 = 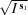. For the initial guess of the object’s phase, three options were explored: (i) a phase grid with a constant value of 0 across the plane [*i.e.* 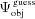 = 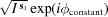]; (ii) a phase grid generated with statistically random values that fluctuate uniformly in the range 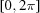 [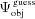 = 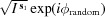]; (iii) a phase grid constructed using the relation between intensity and phase based on the arguments made by Paganin *et al.* (2002 ▸); that is,
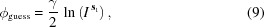
where the ratio 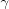 = 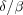 relates to the object’s complex refractive index distribution 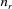 = 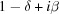. A key assumption of this relation is that the value of γ is constant throughout the object’s volume predicating it is largely composed of a single material. To test the effectiveness of each guess, the reconstruction of 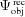 was attained after 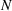 = 1, 10, 100 and 1000. The respective results are shown in Fig. 4 ▸.

In all three cases, the amplitude reconstructions presented in Fig. 4 ▸ require fewer iterations to reach an acceptable solution in comparison with the phase. This is primarily due to the understandably close resemblance of the initial guess to the true amplitude of the exit wavefield at the object. We additionally note that the speckle-like noise pollution for the random phase guess below 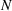 = 100 is likely due to the strong phase gradients being manifested in the intensity, and dis­appears by 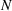 = 1000.

**Figure 5:**
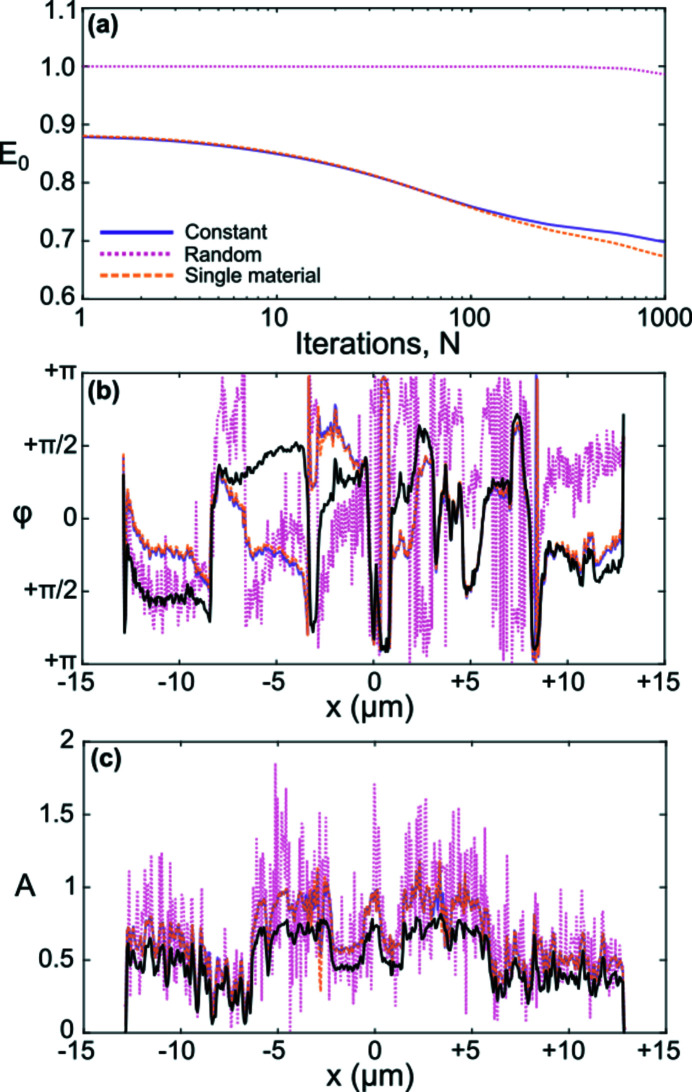
(*a*) Plot showing the evolution of 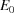 versus number of iterations *N* for all linitial phase guess choices. (*b*, *c*) Display overlaid profiles taken horizontally across the center of the reconstructed phase and amplitude images for 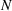 = 1000. The black curve corresponds to the original (true) phase and amplitude input map.

The choice of 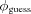 clearly has a decisive effect on the quality and convergence rate of the final phase reconstructions, shown in Fig. 5 ▸. Both the convergence plot [Fig. 5(*a*) ▸] and the quantitative accuracy of the amplitude and phase [Figs. 5(*b*) and 5(*c*) ▸] strongly favor the flat phase or single material assumption over the random phase guess. This is a somewhat surprising observation, as the test phase image (peppers) contained large variations over 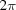, including significant phase gradients. These large phase gradients appeared to cause some significant errors in the recovered phase related to phase-wrapping, though in general the recovered phase is quantitatively similar to the phase of the original test image. More surprising, however, is the improved convergence rate of the single material assumption from 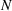 > 200, given that there was no correlation between the phase and amplitude of the test image, which undoubtedly violates its key premise in equation (9). This observation supports recent work by Gureyev *et al.* (2015 ▸), which showed that the single material assumption can extend to a broader class of samples without significant loss of generality. For further details, the reader is encouraged to refer to Gureyev *et al.* (2015 ▸).

## 5. Discussion and conclusion   

Lens translation imaging provides a practical approach to synthetically increasing the numerical aperture, spatial bandwidth product and phase sensitivity of classical full-field hard X-ray microscopes. The methodology is described here in detail using coherent scalar wave optics theory to derive a generalized mathematical expression for the wavefield as it traverses the entire LTI system, and includes a formulation of the iterative phase-retrieval algorithm based on the popular E-PIE algorithm.

In addition to providing the mathematical framework for developing simulation and image recovery code, the analytical expressions also provide valuable physical insights into the image contrast mechanism. In particular, we note that the forward simulations [based on equation (5)] suggest that the off-axis intensity images contain clear contrast in the form of differential phase contrast (DPC). By paying specific attention to the lens amplitude function 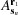, we note the term 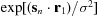 arises once the squared binomial 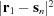 is expanded. Taylor approximating this term to the first order and then invoking the Fourier derivative theorem explains the origin of the DPC signal and how its contribution is proportional to the shifting 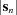. From this it becomes clear that this type of contrast is the same as that observed and studied in visible-light FPM setups (Ou *et al.*, 2013 ▸).

Realizing LTI means that the compound image must be recovered without access to the true amplitude and phase maps as benchmarks for convergence, as was the case here in equation (8). To this end, we recommend calculating the error metric relative to the measure images taken at the various lens positions via the following formula,
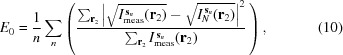
where 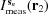 is the measured intensity for a certain 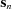, and 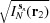 is the calculated intensity at the same 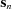 for a particular iteration *N*. The above error metric takes into consideration the average over all lens positions.

Our study of the iterative phase recovery revealed the importance of the initial guess on the rate of convergence and the ultimate quality of the compound image. Like FPM, LTI has an extremely significant advantage that the central image may be used as an accurate guess for the amplitude. However, although the single-material assumption offers some advantages of a constant phase guess, we believe there is still room for improvement. Given that elements of DPC are clearly present in the off-axis images, it seems intuitive that this information could be utilized to provide a more accurate guess of the object phase that would significantly improve the convergence rate.

As a full-field X-ray imaging technique, LTI might have the potential to offer a significantly reduced radiation dose rate (as opposed to accumulated dose) in comparison with scanning-probe methods, such as conventional X-ray ptychography. While the mechanisms of radiation damage vary greatly between specimens, many have clear dependencies on the radiation dose rate (Berejnov *et al.*, 2018 ▸). In the typical cases of a scanning nanoprobe with a 200 nm probe diameter and a full-field microscope with a 200 µm diameter, one could anticipate a reduction in dose rate of six orders of magnitude. In the case of the latest generation of high-brilliance coherent X-ray sources (Eriksson *et al.*, 2014 ▸), this reduction may be an important consideration. On the other hand, the attenuation of X-rays by the objective lens implies that LTI will have a higher accumulated radiation dose than conventional (*i.e.* ‘lensless’) X-ray ptychography for a given field of view and spatial resolution. However, we speculate that this disadvantage may be mediated to an extent by reducing exposure times at small scattering angles, where the intensity is high.

Perhaps most importantly, however, LTI has the potential to be both intuitive and widely accessible at synchrotron beamlines. By retaining the ‘what you see is what you get’ character of full-field microscopy, it provides users with the ability to quickly and decisively design and perform measurements, and to seamlessly switch from fast overviews of the entire specimen (narrow scan range) to detailed inspections of individual elements (broad scan range). Because LTI is based on the classical Galilean geometry used by full-field microscopes around the world, it can also be implemented with little to no additional hardware. Given the imminent improvements in brilliance and coherence of synchrotron sources, we believe LTI could be a convenient, valuable and effective new tool for the broad spectrum of X-ray microscopists.
